# Exploring the Impact of Hand Dominance on Laparoscopic Surgical Skills Development Using Network Models

**DOI:** 10.3390/jcm13041150

**Published:** 2024-02-18

**Authors:** Saiteja Malisetty, Elham Rastegari, Ka-Chun Siu, Hesham H. Ali

**Affiliations:** 1 College of Information Science & Technology, University of Nebraska at Omaha, Omaha, NE 68182, USA; hali@unomaha.edu; 2 Business Intelligence & Analytics Department, Creighton University, Omaha, NE 68178, USA; elhamrastegari@creighton.edu; 3 Department of Health & Rehabilitation Sciences, University of Nebraska Medical Center, Omaha, NE 68198, USA; kcsiu@unmc.edu

**Keywords:** laparoscopic surgery, surgical simulation, hand dominance, learning progression, network models, NASA-TLX scores

## Abstract

Background: Laparoscopic surgery demands high precision and skill, necessitating effective training protocols that account for factors such as hand dominance. This study investigates the impact of hand dominance on the acquisition and proficiency of laparoscopic surgical skills, utilizing a novel assessment method that combines Network Models and electromyography (EMG) data. Methods: Eighteen participants, comprising both medical and non-medical students, engaged in laparoscopic simulation tasks, including peg transfer and wire loop tasks. Performance was assessed using Network Models to analyze EMG data, capturing muscle activity and learning progression. The NASA Task Load Index (TLX) was employed to evaluate subjective task demands and workload perceptions. Results: Our analysis revealed significant differences in learning progression and skill proficiency between dominant and non-dominant hands, suggesting the need for tailored training approaches. Network Models effectively identified patterns of skill acquisition, while NASA-TLX scores correlated with participants’ performance and learning progression, highlighting the importance of considering both objective and subjective measures in surgical training. Conclusions: The study underscores the importance of hand dominance in laparoscopic surgical training and suggests that personalized training protocols could enhance surgical precision, efficiency, and patient outcomes. By leveraging advanced analytical techniques, including Network Models and EMG data analysis, this research contributes to optimizing clinical training methodologies, potentially revolutionizing surgical education and improving patient care.

## 1. Introduction

Surgical simulation training has become increasingly popular over the years as it provides a safe environment for medical professionals to gain experience and improve their skills without putting patients at risk [1,2]. Laparoscopic surgery, a minimally invasive surgical technique, requires specialized skills and hand-eye coordination [3]. The use of simulation training for laparoscopic surgery has been widely adopted as it allows trainees to develop their psychomotor skills and improve their overall performance in a controlled and safe environment [4].

One of the major challenges in surgical simulation training is to develop effective methods to assess the learning progression of trainees. Traditional methods of assessment rely on subjective evaluations by expert surgeons and can be time-consuming and costly [5,6]. The most used method of assessing performance in laparoscopic surgical simulation tasks is to use objective measures such as time to complete a task or error rates [7]. While these measures provide valuable information about trainees’ performance, they do not provide a complete picture of the learning progression of trainees in laparoscopic surgical simulation tasks [8,9]. There is a need for new methods to assess the learning progression of trainees in laparoscopic surgical simulation tasks. Such methods should consider the subjective experience of the trainees themselves, as well as objective measures of performance. The inclusion of subjective measures of performance is particularly important in laparoscopic surgery, where the subjective experience of the participant can impact their performance [10,11]. Additionally, laparoscopic surgery requires the use of both dominant and non-dominant hands to manipulate instruments and perform surgical tasks. It is essential to assess the learning progression of both hands to ensure that surgical trainees develop the necessary skills and proficiency [12,13].

In recent years, there has been a growing interest in using objective and quantitative methods to assess the learning progression of trainees. Network Models have emerged as a promising approach for analyzing and visualizing complex data [14,15]. The use of Network Models to assess the learning progression of laparoscopic surgical simulation tasks is a novel approach that provides a more comprehensive and objective assessment of surgical skills. Network Models can represent complex interactions between different components of a surgical task and can provide valuable insights into the development of surgical skills over time [16]. This research paper proposes a novel assessment method utilizing Network Models to evaluate the learning progression of 20 trainees performing a set of laparoscopic surgical simulation tasks using their dominant and non-dominant hands. The proposed model is populated using the participants’ electromyography (EMG) data, which is used to track their muscle activity during the task performance. Additionally, the NASA Task Load Index (NASA-TLX) score is used to analyze participants’ subjective task demands, allowing for an examination of the impact of participants’ perception of their mental demand, physical demand, temporal demand, performance, effort, and frustration on their simulation task performance.

EMG measures the electrical activity of muscles, providing a quantitative measure of muscle activation and fatigue [17,18]. EMG data has been used to assess the performance of surgical trainees in various surgical procedures, including laparoscopic surgery [17,19]. By analyzing EMG data, the assessment method can identify areas where surgical trainees may need further training or support to improve their surgical skills [20,21,22]. The use of the NASA-TLX score to analyze participants’ subjective task demands is a valuable addition to the assessment method by including insights into the psychological factors that can affect surgical performance [23,24,25].

Assessing both dominant and non-dominant hand use in laparoscopic surgery is important since it is a critical aspect of surgical performance [26,27,28]. Previous research has shown that the use of the non-dominant hand can significantly impact surgical performance, and the development of skills with the non-dominant hand can improve overall surgical proficiency [29,30]. The assessment method developed in this research can provide valuable insights into the learning progression of surgical trainees in laparoscopic surgery, particularly in the development of skills with both dominant and non-dominant hands. The use of Network Models and EMG data can provide an objective and comprehensive assessment of surgical skills [16], this assessment method has the potential to improve the training and support provided to surgical trainees, leading to better patient outcomes.

Additionally, the clinical significance of refining laparoscopic training cannot be understated. Enhanced training protocols, informed by objective assessments of hand dominance and skill acquisition, have the potential to directly impact surgical efficiency, reduce operative times, and minimize the likelihood of complications. By advancing our understanding of how surgeons develop and refine their skills, particularly in manipulating instruments with both hands, we can tailor educational interventions that better prepare surgical trainees for the demands of minimally invasive procedures. Ultimately, this leads to safer surgical practices, improving patient outcomes and aligning with the goals of patient-centered care. The development and application of the proposed assessment method, therefore, not only contribute to the academic and practical realms of surgical education but also promise substantial benefits to the quality of surgical care delivered to patients.

### Problem Statement

The assessment of learning progression and proficiency in surgical simulation training presents a substantial challenge, as current methods relying on subjective evaluations or limited objective measures fail to provide a comprehensive understanding of trainees’ performance [5,6,7]. Moreover, the assessment of both dominant and non-dominant hand proficiency and the consideration of subjective workload often overlooked. Consequently, this study aims to overcome these limitations by introducing an innovative assessment method that integrates objective performance measures, subjective workload evaluation, and analysis of both hand proficiencies. By adopting this comprehensive approach, the study aims to offer a holistic assessment of surgical trainees’ learning progression. The proposed method would inform targeted training interventions and enhance proficiency outcomes in surgical education.

## 2. Materials and Methods

### 2.1. Subjects

We recruited twenty participants (8 female and 12 male) from various disciplines. However, due to incomplete data on performances, participants 19 and 20 were excluded from the analysis. The final analysis included eighteen participants, consisting of nine medical students (or fellows) and nine non-medical students (health professional students in kinesiology, physical therapy, nursing, or radiology). All participants were recruited from the university campus and had no prior experience with the research training simulator or recent upper arm injuries. Each participant confirmed that their right hand was their dominant hand.

### 2.2. Tasks

**Figure 1:**
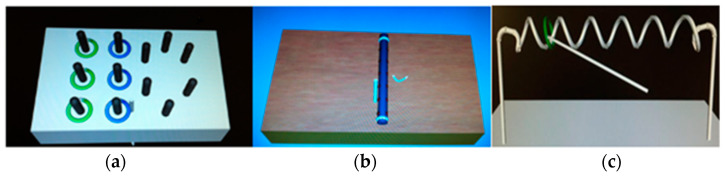
Surgical Simulation Training Tasks: (**a**) Peg Transfer Task (**b**) Needle Passing Task (**c**) Wire Loop Task.

All participants completed a set of basic laparoscopic surgical simulation training tasks, including the peg transfer, needle passing, and wire loop tasks as shown in Figure 1. The peg transfer task involves transferring small pegs from one location to another using laparoscopic instruments, simulating the precision required in surgical procedures. The needle passing task involves manipulating a needle through specific targets using laparoscopic instruments to assess participants’ hand-eye coordination and dexterity. The wire loop task requires participants to maneuver a wire through a series of loops, testing their dexterity and hand-eye coordination.

In this study, we investigated the effect of hand dominance on laparoscopic surgical performance by analyzing the Peg transfer Task and Wire Loop task. While the Needle passing task is a commonly used laparoscopic surgical simulation task, it was excluded from our analysis. The decision to exclude the Needle passing task was based on several considerations. Firstly, our primary research focus was to explore the influence of hand dominance on tasks that inherently require differentiated hand functions. The Needle passing task may not exhibit significant differences in hand requirements, where proficiency can be achieved using either the dominant or non-dominant hand [16]. By focusing on the Peg transfer Task and Wire Loop task, we can provide a targeted investigation into the impact of hand dominance on these specific tasks, shedding light on the role of hand dominance in laparoscopic surgical performance. This approach enhances the precision of our study’s findings and contributes to a comprehensive understanding of how hand dominance affects surgical skill development.

### 2.3. Experiment Setup

The experiment spanned a 4-week period and consisted of one pretraining test, three training sessions, and one post-training test. Participants completed the full set of basic training tasks in each session, with each task repeated five times. Data on performance were collected during three sessions: a baseline session, a session one week after baseline, and a session four weeks after baseline. Following the completion of each task, participants provided feedback on their physical and mental demands using NASA-TLX scores.

### 2.4. Data Collection

Ethical considerations were strictly adhered to in line with the Declaration of Helsinki, receiving approval from the Institutional Review Board of the University (IRB # 103-12-EX). Our study employed the Trigno Wireless System [16], for surface electromyography (EMG) to record muscle activities, focusing on critical muscles including the Biceps Brachii, Triceps Brachii, Extensor Digitorum, and Flexor Carpi Radialis. EMG sensors were meticulously positioned to ensure accurate data capture. The raw EMG signals, acquired at a 2000 Hz sampling rate and band-pass filtered from 20 to 300 Hz, were processed with a root-mean-square (RMS) technique using a 150-ms moving window, enabling precise computation of muscle activation and fatigue.

To mitigate inter-subject variability and enhance the reliability of our EMG data analysis, maximal voluntary contraction (MVC) measurements were obtained from each muscle group. This standardization allowed for the normalization of EMG signals, providing a consistent baseline for comparing muscle activity levels across participants. Additionally, kinematic data, including task completion time, speed, and distance, were meticulously collected for each task, offering quantitative measures of task performance.

Upon the completion of each simulation task, participants engaged with the NASA Task Load Index (TLX) survey, chosen for its robust ability to assess subjective workload across six dimensions: mental demand, physical demand, temporal demand, performance, effort, and frustration. The NASA TLX, developed by Hart and Staveland [23], remains a validated and versatile instrument in various domains, including healthcare and surgical training [25]. Its adoption in our study highlights our dedication to exploring the multifaceted experiences of trainees, aiming to uncover the cognitive and physical challenges encountered during laparoscopic surgery training. Scores from the NASA TLX, ranging from 0 to 10, offered nuanced insights into the subjective workload, with higher scores indicating greater perceived effort.

The integration of detailed EMG data analysis with the multidimensional assessment provided by the NASA TLX underscores the comprehensive nature of our research methodology. This combination offers a holistic examination of the physiological and cognitive dimensions influencing surgical skill development. By capturing objective muscle activity alongside subjective workload perceptions, our approach aims to optimize surgical training protocols. This, in turn, is designed to enhance the efficacy of skill acquisition, contributing to improved surgical performance and patient care outcomes, thereby addressing both the immediate and long-term needs of surgical education.

### 2.5. Network Models

In this study, we utilized Network Models to analyze and evaluate the learning progression of laparoscopic surgical simulation tasks. Network Models are mathematical representations that consist of nodes and edges, where nodes represent entities or elements, and edges represent relationships or connections between those entities.

#### 2.5.1. Nodes and Edges

In our Network Models, nodes represent the individual participants who engaged in the laparoscopic surgical simulation tasks. Each node corresponds to a unique participant and can be enriched with additional information, such as demographic data, experience level, or performance metrics. On the other hand, edges in our study serve to capture the connections or relationships between these participants. However, our approach diverges from examining edges within the same network of participants. Instead, we focus on comparing the similarity of networks formed across different sessions. These edges signify the presence or absence of connections between networks established in various sessions, enabling us to explore changes and patterns of connectivity over time. By emphasizing the inter-session comparisons, our study unveils the dynamic nature of skill acquisition and development, shedding light on how participants’ interaction patterns evolve throughout their training journey.

#### 2.5.2. Network Density

Network density, a fundamental measure in graph theory, was employed to assess the overall connectivity and complexity of hand movements during the laparoscopic simulation tasks. It is defined as the ratio of the number of actual edges in the network to the total possible number of edges [31]. A higher network density indicates a greater level of interconnectedness among participants’ hand movements, suggesting increased coordination and information flow within the network. In our study, network density served as a quantitative indicator of the degree to which participants’ hand movements were interconnected, providing valuable insights into the efficiency in performing laparoscopic surgical simulation tasks. By analyzing network density, we were able to evaluate the structural characteristics and overall efficiency of within the network of hand movements, contributing to a comprehensive understanding of the participants’ performance and skill development in the simulation environment.

#### 2.5.3. Comparing Similarity of Networks Using Jaccard Similarity

To assess the similarity between networks, we employed the Jaccard similarity coefficient. It is defined as the ratio of the number of common edges between two networks to the total number of edges in the two networks [32]. This coefficient measures the extent of overlap between the edges of two networks, indicating the common connections they share as shown in Equation (1). The Jaccard similarity coefficient ranges from 0 to 1, where 0 indicates no similarity and 1 indicates complete similarity between the networks.

J (G1, G2) = |E(G1) ∩ E(G2)|/|E(G1) ∪ E(G2)|(1)

where:

E(G1) represents the set of edges in network G1 for a specific session.

E(G2) represents the set of edges in network G2 for another session.

|E(G1) ∩ E(G2)| denotes the number of common edges between the two networks.

|E(G1) ∪ E(G2)| denotes the total number of distinct edges in both networks.

By applying the Jaccard similarity coefficient to compare networks formed in different sessions, we evaluated changes in the network structure and identified learning progression among participants. A higher Jaccard similarity coefficient suggested a greater similarity in the network structure between sessions, implying consistent patterns in the connections formed at different time points. Conversely, a lower coefficient indicated significant differences in the network structure, indicating learning progression or changes in the connections between networks. Utilizing the Jaccard similarity coefficient provided valuable insights into the evolution of network structure and the development of consistent patterns or changes in muscle movement similarities among the surgical trainees across different sessions. This analysis allowed us to quantify the degree of change or similarity in network connections and further understand the learning dynamics in laparoscopic surgical simulation tasks.

#### 2.5.4. Global Clustering Coefficient

The global clustering coefficient assesses the extent to which nodes in the entire network tend to form clusters or groups. It measures the overall cohesiveness or clustering in the entire network. A global clustering coefficient of 0 suggests that nodes in the network are not organized into clusters whereas a coefficient of 1 indicates that the network has a high level of clustering, with many nodes forming tightly interconnected groups [33].

In our study, the global clustering coefficient serves as a crucial metric in unraveling the intricacies of hand movement dynamics during laparoscopic surgical simulation tasks. This coefficient quantifies the tendency of participants’ hand movements to cluster into cohesive groups within the network. A high clustering coefficient indicates that participants are more likely to exhibit similar hand movement patterns, forming distinct clusters within the network. Such clusters could signify specific groups of participants who share similar strategies or skill development trajectories. Understanding the clustering coefficient within our network sheds light on how participants’ hand movements tend to synchronize and coordinate, highlighting the presence of subgroups or communities within the larger network. This insight not only enriches our comprehension of the structural characteristics of hand movement networks but also provides valuable information regarding skill acquisition patterns among surgical trainees. Ultimately, the clustering coefficient serves as a critical tool in deciphering the intricate learning dynamics that underlie the development of laparoscopic surgical skills.

#### 2.5.5. Modularity Coefficient

Modularity Coefficient quantifies the quality of the partitioning of our network into modules or communities [34]. A positive modularity score signifies the presence of significant and distinct communities within the network, where participants within a community share similar hand movement patterns. In our study, the Modularity Coefficient measures the extent to which our participants’ hand movement networks can be subdivided into distinct, internally well-connected clusters. A higher Modularity Coefficient suggests a stronger community structure within the network, indicating that participants’ hand movements tend to form cohesive groups during the laparoscopic simulation tasks. By evaluating modularity, we gain a deeper understanding of how the interactions and dependencies between participants’ hand movements evolve throughout the training process. This analysis enables us to identify potential patterns of coordination and cooperation among participants, shedding light on the dynamics of skill acquisition in laparoscopic surgical simulation tasks.

#### 2.5.6. Network Model Creation and Comparative Analysis

**Figure 2:**
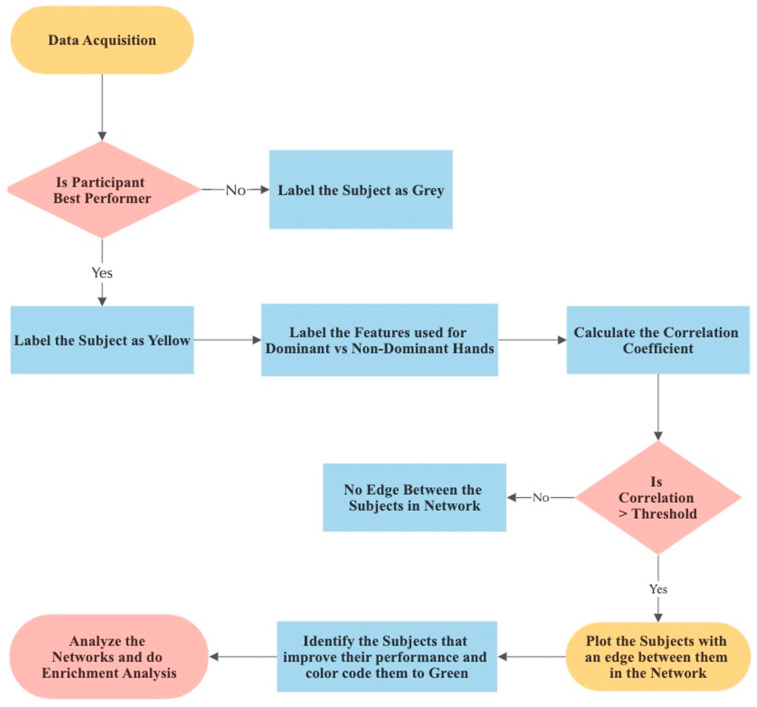
Flow Chart of Network Model Creation.

In this study, we utilized a network-based approach as shown in Figure 2 to analyze the correlations among participants’ electromyographic (EMG) data, allowing us to explore the similarity and relationships between individuals. Each participant was represented as a node in the correlation network graph, and edges were created to connect nodes that exhibited a strong correlation in their EMG features.

To assess the participants’ similarity, Pearson’s pairwise correlation coefficient (ρ) is used, measuring the linear dependence between their EMG features. A correlation coefficient of 0 indicates no meaningful relationship, while a coefficient of 1 represents an extremely strong association.

(2) $$\mathrm{SM}\;(i,j)=\left\{\begin{array}{l} 1,\;\mathrm{if}\;(\rho (\mathrm{Pi},\mathrm{Pj}))\;\geq \;k\\ 0,\;For\;other\;Cases \end{array}\right.$$

By calculating the correlation coefficients for each pair of participants, a correlation matrix (CM) is generated, reflecting the strength of the relationships. The correlation criterion “k” is chosen to determine the level of similarity among participants. By solving the Equation (2) and setting a threshold (e.g., 90%), a significance matrix (SM) is created, representing the adjacency matrix of the correlation network graph. If SM [i, j] is 1, then subjects i and j are linked in the network or else there is no edge or link between the subjects.

To enhance the visualization of our observations and facilitate a better understanding of participant performance, we introduced color-coded nodes in the network. The purpose of this color-coding was to highlight specific participants who demonstrated superior performance during a critical phase of the study. We chose to identify the best-performing participants based on their performance in the fifth trial of the third session.

There are several reasons behind this choice:

1. Stability of Skill: By the time participants reached the fifth repetition of the third session, they had gained considerable experience and practice with the task. This phase represents a more stable level of skill compared to earlier repetitions, where participants might still be improving and adapting to the task.
2. Consistency: Selecting the fifth repetition ensures that participants had repeated the task multiple times, which allows us to observe their performance consistency. Consistency is an essential factor in skill assessment and is often more informative than isolated exceptional performances.
3. Focused Analysis: By focusing on the last session and repetition, we narrow our analysis to the participants’ most recent and presumably refined skill levels. This helps us pinpoint participants who have achieved a high level of proficiency by the end of the study.
4. Practical Application: In real-world scenarios, such as surgical training or other skill-based tasks, it is often essential to identify individuals who consistently perform well in critical moments. Emphasizing the last session’s task completion time helps identify participants who can maintain superior performance even under demanding circumstances.
5. Comparison Potential: By comparing the best-performing participants with others in the network, we can gain insights into the characteristics or behaviors that distinguish their superior performance. This comparison may provide valuable information for identifying factors that contribute to overall success.

In conclusion, selecting the best-performing participants based on their task completion time in the fifth repetition of the third session allowed for a focused analysis, providing insights into skill stability, consistency, and factors contributing to exceptional performance. Furthermore, we expanded our analysis by color-coding nodes of participants who exhibited improvement over time and shared similar hand movements with the best performers, highlighting their progression. To investigate the impact of hand dominance on learning, we constructed separate networks using dominant and non-dominant hand features. This enabled us to evaluate which hand features better reflected participants’ learning progression. By integrating these additional elements into the network model, we aimed to gain deeper insights into the relationship between participants’ performance, hand dominance, and the corresponding network structures.

## 3. Results

**Figure 3:**
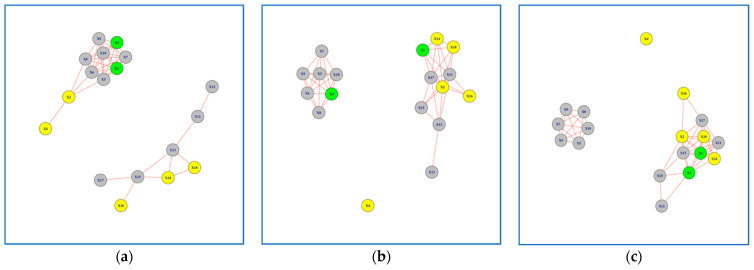
Network Models for Peg Transfer Task using Non-Dominant Hand Features: (**a**) network model in Session 1; (**b**) network model in Session 2. (**c**) network model in Session 3. Subjects with superior performance over others are colored in Yellow, whereas subjects who improved performance over time are colored in Green and the rest are colored in Grey.

**Figure 4:**
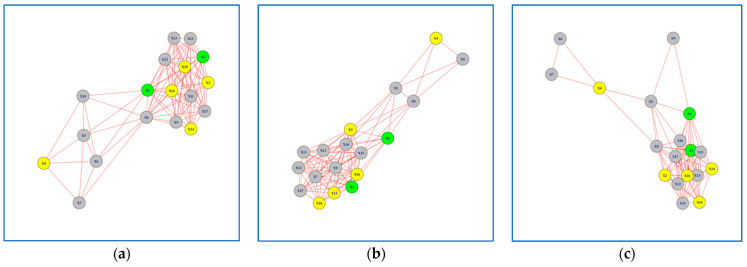
Network Models for Peg Transfer Task using Dominant Hand Features: (**a**) network model in Session 1; (**b**) network model in Session 2. (**c**) network model in Session 3. Subjects with superior performance over others are colored in Yellow, whereas subjects who improved performance over time are colored in Green and the rest are colored in Grey.

**Figure 5:**
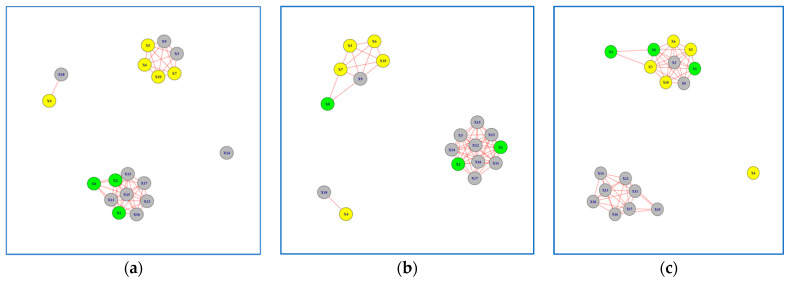
Network Models for Wire Loop Task using Non-Dominant Hand Features: (**a**) network model in Session 1; (**b**) network model in Session 2. (**c**) network model in Session 3. Subjects with superior performance over others are colored in Yellow, whereas subjects who improved performance over time are colored in Green and the rest are colored in Grey.

**Figure 6:**
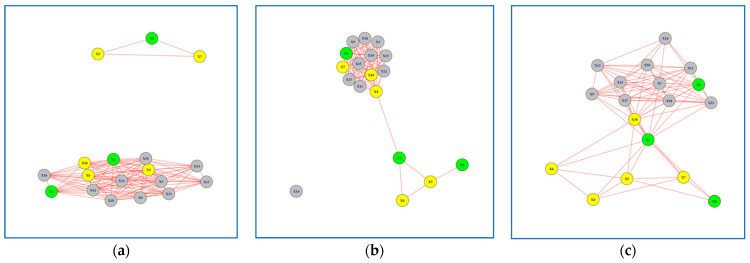
Network Models for Wire Loop Task using Dominant Hand Features: (**a**) network model in Session 1; (**b**) network model in Session 2. (**c**) network model in Session 3. Subjects with superior performance over others are colored in Yellow, whereas subjects who improved performance over time are colored in Green and the rest are colored in Grey.

In this section, we present the 12 networks created based on the results of Peg Transfer and Wire Loop tasks. For the Peg Transfer task, participants performed the task using both their dominant and non-dominant hands. We formed six networks for each task (Figure 3, Figure 4, Figure 5 and Figure 6), with three networks utilizing dominant hand features and three networks utilizing non-dominant hand features. These networks were created based on the EMG values of each session.

### 3.1. Networks for Peg Transfer Task

The examination of the Peg Transfer task networks revealed interesting patterns, particularly for Subjects 1 and 3, which were highlighted in green. These subjects exhibited a clear learning progression when analyzing their non-dominant hand movements (Figure 3a–c). The Jaccard Similarity, a measure of network overlap, between networks formed in Session 1 versus Session 3 was 0.30 for the non-dominant hand and 0.63 for the dominant hand (Table 1). This indicates that the networks formed in Session 3 differ by 70% when compared to the networks formed in Session 1 for the non-dominant hand. Conversely, for the dominant hand, the difference was only 27%, suggesting a higher level of consistency in network formation between sessions. It is worth noting that the learning progression of Subjects 1 and 3 was not as evident when using the dominant hand features to build the network model (Figure 4a–c).

Although the best performers were identified using the task completion time in the fifth trial of the third session, subject 4 is not connected to the other best performers in the network model (Figure 3a–c). This is due to a different muscle activity pattern for subject 4 compared to the rest of best performers. Looking at the completion time, we noticed that subject 4 took much less time compared to the rest, meaning that this subject is outperforming the others. Aside from analyzing each network in Figure 3, it is also worth analyzing each sub-network. As it appears in the network models, there are two sub-networks. If the subjects are learning how to perform the task and becoming closer to the best performers, then the expectation would be to see a change in the density of sub-networks such that one gains more edges over time indicating that there are more subjects with similar performance to the best performers. This pattern is exactly what we see in Figure 3. We can see that over time the sub-network from Figure 3a, 3b, to 3c that include more yellow nodes compared to the other sub-network, has more edges and consequently a higher density, indicating that more subjects perform similar to best performers as they practice over the training sessions.

In analyzing the Peg Transfer task networks, various network model parameters were assessed as shown in Table 2. All networks shared a common composition of 18 nodes, but significant disparities emerged when comparing dominant and non-dominant hand networks. Dominant hand networks consistently boasted a higher number of edges (94, 97, and 83 for Sessions 1, 2, and 3) and network density (0.61, 0.63, and 0.54) in contrast to non-dominant hand networks (edge counts of 41, 46, and 49 and network densities of 0.26, 0.30, and 0.32). Further examination of clustering coefficients unveiled that non-dominant hand networks consistently exhibited denser local clusters (0.72, 0.69, and 0.73) compared to their dominant hand counterparts (0.51, 0.55, and 0.57).

Furthermore, modularity values exhibited positive trends across Sessions 1, 2, and 3 for both non-dominant (0.49, 0.41, and 0.46) and dominant hand networks (0.19, 0.15, and 0.18). These positive values indicate the presence of distinct communities within the networks. However, it’s noteworthy that the tendency to form such communities appears weaker in the case of dominant hand networks, as evidenced by their lower modularity coefficients. These results provide valuable insights into the learning progression and differences between the dominant and non-dominant hand networks in peg transfer task.

### 3.2. Networks for Wire Loop Task

For the Wire Loop task, participants were instructed to first perform the task using their dominant hand (WR) and then repeat it using their non-dominant hand (WL). Similar to the findings in the Peg Transfer task, we observed a distinct learning progression for Subjects 1, 2, and 8, which were highlighted in green, when analyzing their non-dominant hand movements (Figure 5a–c). It is also worth noting that the learning progression of Subjects 1, 2 & 8, highlighted in green, was not as evident when using the dominant hand features to build the network model (Figure 6a–c).

The Jaccard Similarity between Networks formed in Session 1 versus Session 3 was 0.38 for the non-dominant hand and 0.61 for the dominant hand (Table 3). This suggests that the networks formed in Session 3 differed by 62% when compared to the networks formed in Session 1 for the non-dominant hand. Conversely, for the dominant hand, the difference was 39%, indicating a higher level of consistency in network formation between sessions.

Moreover, we conducted a comprehensive analysis of network model parameters for the Wire Loop task networks, which is summarized in Table 4. Across all sessions, the networks consistently comprised 18 nodes. Specifically, the dominant hand networks exhibited edge counts of 104, 83, and 83 for Sessions 1, 2, and 3, respectively. In contrast, the non-dominant hand networks had edge counts of 57, 49, and 55 for Sessions 1, 2, and 3, respectively. Examining network density, we observed that the dominant hand networks recorded values of 0.67, 0.54, and 0.54 across the three sessions, while the non-dominant hand networks displayed densities of 0.37, 0.32, and 0.35. This pattern echoes the trends observed in the Peg Transfer task, where networks formed using the dominant hand consistently exhibited a higher number of edges and greater network density compared to their non-dominant counterparts.

A closer examination of clustering coefficients in the wire loop task revealed a consistent trend. Non-dominant hand networks consistently displayed denser local clusters (0.69, 0.62, and 0.78) compared to their dominant hand counterparts (0.49, 0.42, and 0.37). Additionally, when considering modularity values across Sessions 1, 2, and 3, we observed similar positive trends for both non-dominant (0.54, 0.47, and 0.59) and dominant hand networks (0.29, 0.23, and 0.21). These positive values signify the existence of distinct communities within the networks. Nonetheless, it’s worth noting that the tendency to form such communities appears less pronounced in the case of dominant hand networks, as indicated by their comparatively lower modularity coefficients.

These results offer valuable insights into the learning progression and disparities observed in the Wire Loop task networks. The pronounced learning patterns exhibited by Subjects 1, 2, and 8, highlighted in green, when analyzing their non-dominant hand movements underscore the importance of considering hand dominance in performance assessment. Our analysis of network model parameters further substantiates these findings, revealing variations in edge counts and network density between dominant and non-dominant hand networks.

In summary, our study emphasizes the significance of analyzing non-dominant hand movements, particularly in tasks involving both hands, for distinguishing participants with varying levels of learning proficiency. The disparities observed in networks formed during different sessions provide valuable insights into the learning progression of participants undertaking the Peg Transfer and Wire Loop tasks.

### 3.3. Enrichment Analysis with NASA-TLX Scores

Enrichment analysis was employed in this study to gain deeper insights into the relationship between subjective workload, perceived task demands, and participants’ learning progression and performance in laparoscopic surgical tasks. This statistical technique allowed us to explore the associations and patterns between the NASA-TLX scores and participants’ outcomes. By comparing the scores of participants with different learning progression levels or performance outcomes, we were able to identify potential factors that contribute to enhanced learning and performance. The NASA-TLX assesses subjective workload and perceived task demands experienced by individuals, including mental demand (MD), physical demand (PD), temporal demand (TD), performance, effort, and frustration. Scores for each dimension range from 0 to 10, with 10 indicating the highest subjective effort.

For the Peg Transfer task, participants who demonstrated a higher learning progression, such as Subjects 1 and 3, reported elevated physical demand average scores (e.g., PD = 7.2) indicating a higher perceived physical effort required to perform the task. Additionally, the best performers in the Peg Transfer task, highlighted in yellow, also reported relatively high physical demand average scores (e.g., PD = 6.8) compared to other participants. These findings suggest that a higher physical demand may be associated with enhanced learning and performance.

In terms of mental demand, participants who exhibited a significant learning progression in both the Peg Transfer and Wire Loop tasks reported lower mental demand average scores (e.g., MD = 3.5 for Peg Transfer, MD = 4.2 for Wire Loop) indicating a reduced perceived cognitive effort. Similarly, the best performers in both tasks reported relatively lower mental demand average scores (e.g., MD = 3.2 for Peg Transfer, MD = 4.0 for Wire Loop). These findings suggest that a lower mental demand may contribute to improved learning progression and performance.

Regarding temporal demand, participants’ scores indicated a moderate perceived pressure associated with task time constraints. However, no significant correlations were observed between TD and learning progression or the identification of best performers in either task. Furthermore, participants’ effort scores demonstrated a positive correlation with learning progression. Subjects who exhibited a higher learning progression reported increased effort average scores (e.g., Effort = 6.5) indicating a greater investment of energy and motivation. The best performers in both tasks also reported relatively high effort average scores (e.g., Effort = 6.2 on average). These findings suggest that participants who exerted more effort during the tasks were more likely to show improved learning and performance. Interestingly, frustration scores did not demonstrate a strong correlation with learning progression or the identification of best performers in either task. Participants reported moderate levels of frustration (e.g., Frustration = 4.0) across all sessions, suggesting that frustration may not have significantly impacted learning progression or performance.

Overall, our enrichment analysis with NASA-TLX scores supports that specific dimension of task demands, such as physical demand, mental demand, and effort, are associated with participants’ learning progression and the identification of best performers. Higher physical demand and effort, combined with lower mental demand, appear to be linked to enhanced learning and performance. These findings underscore the importance of considering task demands and subjective experiences in assessing participants’ performance and designing effective laparoscopic surgical training programs.

## 4. Discussion

Our study presents a novel assessment method utilizing Network Models to evaluate the learning progression of laparoscopic surgical simulation tasks. By analyzing participants’ electromyography (EMG) data and subjective task demands using the NASA-TLX scores, we gained valuable insights into the development of surgical skills. This approach holds significant implications for surgical education, providing educators with a comprehensive understanding of trainees’ performance and needs.

The effectiveness of Network Models in assessing surgical skills was evident in our study. The visualization of networks allowed for a comprehensive evaluation of performance by capturing complex interactions within the surgical task performance. This objective and quantitative approach aligns with the growing interest in utilizing advanced methods for skill assessment in surgical simulation training [16]. By incorporating Network Models, educators can identify trainees who may be lagging, require additional training, or need more supports, enabling targeted interventions to optimize skill development.

The utilization of the NASA Task Load Index (TLX) in our study underscores the importance of considering subjective workload in the evaluation of surgical training efficacy. Despite being developed over three decades ago, the NASA TLX remains a gold standard in workload assessment, owing to its robust validation and adaptability to various research and clinical settings [23,24,25]. By incorporating the NASA TLX, we gained valuable insights into the specific challenges and demands experienced by trainees, informing the design of more nuanced and effective training protocols that cater to the cognitive and physical capacities of learners.

Our findings emphasized the importance of assessing the learning progression of both dominant and non-dominant hands in laparoscopic surgery. The non-dominant hand plays a crucial role in supporting the camera or manipulating tissue, and the development of proficiency with this hand is essential for overall surgical performance [26]. By considering the learning progression of both hands, our assessment method ensures a well-rounded development of skills among surgical trainees. This insight can guide educators in designing training programs that address the specific needs of trainees in developing proficiency with both hands.

The variability in EMG responses among participants, especially when performing complex manipulations, presents a methodological challenge that we addressed through careful data analysis and interpretation. By normalizing EMG data to individual MVC values, we minimized the impact of physiological differences on our results, allowing us to focus on variations in muscle activity patterns related to learning progression and task performance. Furthermore, our analysis considered the specific demands of laparoscopic surgical tasks, enabling us to distinguish between skill-related changes in muscle activity and variability stemming from individual differences. Our study’s emphasis on result-oriented analysis of EMG data underscores its potential to inform targeted training interventions. By identifying specific muscle activity patterns associated with successful task performance, we provide actionable insights that can be used to customize training programs, addressing the unique needs of each trainee. This approach not only enhances the precision of skill assessment in laparoscopic surgery but also contributes to the development of more effective, personalized training methodologies.

The calculation of Jaccard similarity provided valuable insights into the differences in network formation between sessions, particularly for the non-dominant hand. The significant divergence in network formation between session 1 and session 3 indicated substantial learning progression, highlighting the effectiveness of our assessment method. In contrast, the dominant hand showed a lower difference in network formation, suggesting that trainees exhibit more consistency in their performance with their dominant hand. These comparative analyses provide educators with a deeper understanding of trainees’ skill development and can inform targeted interventions. Overall including the analysis of clustering coefficient and modularity highlights the existence of community structures in the networks, indicating that participants’ hand movements tend to form distinct communities with stronger connections among nodes within the same community. This insight adds another layer of understanding to the organization of hand movements in laparoscopic surgical tasks and underscores the relevance of community detection methods in network analysis.

Our comprehensive assessment method, which combines Network Models, EMG data, and subjective task demands assessment, offers several advantages over traditional methods of skill assessment. While traditional methods rely on subjective evaluations by expert surgeons or objective measures such as task completion time or error rates [5,6,7], our method provides a more holistic and multi-dimensional assessment. It considers objective performance metrics as well as subjective experiences, providing a more comprehensive picture of the learning progression of surgical trainees.

Moreover, our exploration into the use of Network Models and EMG data analysis offers a complementary perspective to the structured, measurable feedback provided by contemporary laparoscopy training systems, such as the DaVinci training complex and LapSim [35,36]. These advanced systems have set a high standard in surgical education by enabling precise assessment of a trainee’s performance across a variety of tasks, thus facilitating immediate and targeted feedback [35,36]. However, by delving into the physiological underpinnings of surgical skill acquisition, particularly the role of hand dominance, our study broadens the scope of surgical training evaluation. It introduces a dimension that goes beyond the immediate feedback on task execution to include an analysis of muscle engagement and neuromotor control, essential factors in the development of surgical competence. This nuanced approach not only enhances our understanding of the complex interplay between physical and cognitive demands in surgical training but also suggests avenues for integrating our findings with existing laparoscopy training technologies. For instance, insights from our method could be utilized to refine the feedback mechanisms of these systems, offering a more holistic view of trainee performance that encompasses both the outcomes of surgical tasks and the physiological strategies employed during task execution. Such integration promises to tailor training more closely to individual needs, thereby optimizing skill development and enhancing the overall efficacy of surgical education. This potential for synergy between traditional training systems and our novel assessment method underscores the value of interdisciplinary approaches in advancing surgical training and ultimately improving patient care.

### 4.1. Limitations

While our study proposes an innovative and comprehensive approach to evaluating the learning progression of students in surgical simulation tasks, there are certain limitations to consider. Firstly, the sample size of 18 participants may limit the generalizability of our findings. A larger sample size would provide a more representative sample and increase the statistical power of our study. Secondly, the use of EMG data may not capture all aspects of the participants’ performance. While EMG data provides valuable data on muscle activity, other factors such as hand-eye coordination, dexterity, and overall skill level may also impact performance. Thirdly, the NASA-TLX score, while useful, relies on self-reporting by participants and may be subject to biases or inaccuracies. Lastly, our research does not directly assess their ability to perform in real-life surgical scenarios. Future research could explore the transferability of skills learned through simulation tasks to actual surgical procedures.

### 4.2. Future Directions

Building on the current study, future research should aim to expand the assessment method to incorporate additional objective measures and subjective evaluations. This could include integrating other physiological data, such as heart rate or eye-tracking, to provide a more comprehensive assessment of surgical performance. Additionally, an exciting avenue for future research is the development of a decision support system based on our assessment methodology. By leveraging the insights gained from Network Models, EMG data, and subjective overload levels, a decision support system could provide real-time feedback and recommendations to surgical trainees. This system could analyze performance patterns, identify areas of improvement, and offer tailored interventions to enhance skill development.

## 5. Conclusions

In this study, we proposed a novel assessment method utilizing Network Models to evaluate the learning progression of laparoscopic surgical simulation tasks. Analyzing participants’ EMG data and subjective task demands using the NASA-TLX score provided us with valuable insights into the development of surgical skills. This method, by capturing the complex interactions between different components of the surgical task and objectively assessing participants’ muscle activation and fatigue, offers quantitative measures of their performance. Furthermore, the inclusion of subjective task demands, assessed through the NASA-TLX score, shed light on the psychological factors that can influence surgical performance.

Our findings underscore the potential of this assessment method to significantly enhance the training and support provided to surgical trainees. By highlighting the learning progression of both dominant and non-dominant hands, this research emphasizes the critical need for developing proficiency in both hands to improve laparoscopic surgery outcomes. This comprehensive approach, grounded in a deep understanding of the physiological and psychological aspects of surgical training, has the potential to transform surgical education. By ensuring that trainees acquire the necessary skills and dexterity, we can ultimately lead to safer surgical procedures and better patient outcomes. The clinical implications of our study extend beyond the academic domain, offering a promising avenue for the application of Network Model-based assessments in surgical simulation tasks. These advancements could revolutionize surgical training, making it more personalized and effective, thereby contributing to the enhancement of patient care in the realm of minimally invasive surgery.

## Figures and Tables

**Table 1:** Comparison of Networks formed in each Session for Peg Transfer Task.

Parameter	Non-Dominant Hand			Dominant Hand		
	Session 1-2	Session 2-3	Session 3-1	Session 1-2	Session 2-3	Session 3-1
Jaccard Similarity	0.35	0.53	0.30	0.59	0.59	0.63

**Table 2:** Network Model Parameters for Peg Transfer Task.

Parameter	Non-Dominant Hand			Dominant Hand		
	Session 1	Session 2	Session 3	Session 1	Session 2	Session 3
Number of Nodes	18	18	18	18	18	18
Number of Edges	41	46	49	94	97	83
Network Density	0.26	0.30	0.32	0.61	0.63	0.54
Clustering Coefficient	0.72	0.69	0.73	0.51	0.55	0.57
Modularity Coefficient	0.49	0.41	0.46	0.19	0.15	0.18

**Table 3:** Comparison of Networks formed in each Session for Wire Loop Task.

Parameter	Non-Dominant Hand			Dominant Hand		
	Session 1-2	Session 2-3	Session 3-1	Session 1-2	Session 2-3	Session 3-1
Jaccard Similarity	0.55	0.42	0.38	0.53	0.58	0.61

**Table 4:** Network Model Parameters for Wire Loop Task.

Parameter	Non-Dominant Hand			Dominant Hand		
	Session 1	Session 2	Session 3	Session 1	Session 2	Session 3
Number of Nodes	18	18	18	18	18	18
Number of Edges	57	49	55	104	83	83
Network Density	0.37	0.32	0.35	0.67	0.54	0.54
Clustering Coefficient	0.69	0.62	0.78	0.49	0.42	0.37
Modularity Coefficient	0.54	0.47	0.59	0.29	0.23	0.21

## Data Availability

Data is contained within the article.
